# Lymphangitis

**Published:** 1897-01

**Authors:** A. B. Campbell

**Affiliations:** Berlin, Ontario, Canada


### LYMPHANGITIS.

By A. B. Campbell, V. S.,
BERLIN, ONTARIO, CANADA.

This subject is old and threadbare in veterinary literature, and I predict it will be passed over by the busy veterinarian with a casual look at the heading; but I hope any who read the results of my experience noted, and whose experience corroborates the same, or if any doubt the validity of my remarks, I hope they will seize the opportunity so kindly afforded us through the columns of this Journal to communicate our thoughts and experiences to each other, and set their pen to work that I may be strengthened or refuted in the views I express. I maintain that from 90 to 100 per cent. of the cases called lymphangitis are not lymphangitis, and if the lymphatics become inflamed it is only secondarily; that the majority, if not all, originate from venous obstruction or thrombus.

The symptoms laid down in our text-books are very plain, so much so that any ordinarily intelligent person reading them would have no trouble in diagnosticating a case should he meet one; but I will take those symptoms one by one and see how I can apply them to my theory. I will follow Professor Robertson, as his work is nearest at hand. He divides the symptoms into "local" and "constitutional." I will give constitutional first place, as the first factor in the causation of disease is a changed or abnormal condition of the blood, which renders it more readily coagulable.

The constitutional or general symptoms are shivering fits, elevated temperature, increased number of pulsations, with a full, tense, cord-like artery; in more severe cases the respirations become hurried, short, and catching, with impairment of appetite, etc. All these general symptoms can be accounted for as a result of the local. In the first place, the animal for some time previous to the attack is accumulating some substance into the blood or the blood is lacking of some of the elements which render it normal. At present I am not able to state the exact condition that exists, but it is doubtless that the blood is in a readily coagulable state. While the animal is at his usual work or is not called upon to perform extra exertion, nothing is noticed unless it is by a careful observer, who will notice the feces dryer, adhering more to the rectum, and the animal has not his usual spirits, is feeling kind of lazy, etc.

Then, after being in active exercise, when the action of the muscles on the valve veins of the limbs has rendered the venous circulation of the limb active, venous blood is almost completely removed from the limbs, then comes enforced rest, the animal is almost motionless, there is a stasis of the venous circulation until arterial pressure is again sufficient to continue the flow. During this period of stasis the blood held above the valves and at points where there is a dilated state of the vein coagulation commences. This point in the hind limb being in the great saphena vein, just before it takes an acute angle over the border of the sartorius muscle to enter the femoral vein. A thrombus once formed, the fibrin is continually adhering to it until the vessel is completely obstructed and the vessels below distended. It is then that our constitutional symptoms commence. The first to be noticed is a full, tense artery, caused no doubt in two ways: first, by a portion of the circulation being obstructed, disturbing the equilibrium of blood between veins and arteries; second, by nervous impulses from the distended portion of the circulation calling on the great motor centre for more power to remove the obstruction. This is simply increased heart-activity, which is the cause of elevated temperature. Now, coming to the local symptoms. I have already accounted for the sudden lameness, although in reality it comes on gradually; swelling and tenderness in the region of the inguinal or bronchial lymphatic glands. I will speak more directly of the inguinal, as we meet it more frequently in that region. From my researches it would be difficult to feel the lymphatic glands of that region if they were nodulated, as they are deeply seated, the nearest being the precrural group. The nodulated condition I find is thrombi, divided by the valves of the vessel. Swelling is the result, and it will naturally commence along the veins which are distended and gradually enlarge the whole limb as high as the level of the thrombus, where it generally terminates abruptly. The intense pain is easily enough accounted for when we consider the force that is constantly on the already distended vessel, with no channel of escape.

I have so far accounted for every symptom that at present presents itself, but if I have omitted any they are equally as easily accounted for. I will now imperfectly describe a couple of cases which came under my notice. My attention was directed strongly to this disease some time ago by a paper on this subject at one of our society meetings, the principal discussion being on the cause, and as the animal which I drive was subject to attacks I resolved to follow it closely.

Case I.—Subject a strong, sixteen-hand bay horse; good limbs and feet well placed; bred from Chicago Volunteer; high temperament and good roadster; at present nine years old. Before coming into my hands had several attacks, and the limb was considerably enlarged, and shortly after had another acute attack, followed by an unaccountable attack of congestion of the lungs, which left a thickness of wind for several months, which would increase on being confined to the stable without plenty of fresh air. At this time I had not thought of thrombi. In February last, when applying a lotion to the inner side of the thigh, as the acute symptoms were abating, I was struck by the hard, pencil-like condition of the superficial veins radiating from the point of the great saphena vein, where it takes an abrupt turn between the muscles to empty into the femoral, at which point was a large, hard lump. I could not define what it was at first, but on careful examination and constantly watching this and other cases I satisfied myself that the veins of the vicinity were filled with thrombi, which gradually absorbed and disappeared. This animal has not had an attack since, as I use preventive measures, viz., an alterative ball about once in two or three months, with occasional small doses of pot. nit. or mag. sulph., and the limb is very little larger than normal, although eighteen months ago it was unsightly, with the skin broken in creases across the front of the hock. The thick wind yielded to treatment with Fowler's solution of arsenic, tonic powders, and cough-balls.

Case II.—Bay horse, six years old; very similar disposition to the animal in Case I. First attack in February last. Was treating at the time the thrombi were disappearing in Case I. This took a similar course. Had a second attack in April and a third September 5th. This attack I will try to describe, although I took no notes. The animal for some time had not been chewing properly; internal parasites suspected, which on subsequent treatment were removed. The day previous to the attack had been driven until pretty tired. September 5th was standing in stable. Before noon was noticed by owner to show symptoms of the old trouble, what we called lymphangitis. I was called and I found him standing, trembling at the flank. Pulse full and about 72. Temperature 103°. Breathing heavy or increased. Anxious expression. When the hand was gently placed on the inner side of the thigh he raised the limb and almost fell over. The superficial veins were enormously distended with a large, hard lump at the angle of the great saphena. The limb was swollen all round up to the hock, and on the inner side up to the lump mentioned. I now decided to find the nature of the trouble. I inserted a needle into the distended vessel, and venous blood escaped. This satisfied me that it was the venous and not the lymphatic circulation that was impeded. I then inserted the needle into the lump, and when withdrawn a portion of the thrombus was contained in the cavity of the needle. Against my better judgment I wished to dislodge this thrombus for further proof. I applied cloths wrung from hot water and then manipulated, when the thrombus broke in several pieces and passed up with a rush of blood. The animal was immediately relieved, and the swelling abated, and after the effects of the purgative (which was administered first) were over was apparently all right.

It was then that the following dose was given for ascaris megalocephalus: One-half pint of wormwood tea; one-half pint of whiskey; one-half pint of milk; two drachms of tartar emetic. Hundreds of the parasites were passed during the next four days. Being owned by a livery keeper, was sent out on a trip in cold, wet weather, and came home suffering from pleurisy, followed by hydrothorax and death. The post-mortem revealed several pails of fluid in the thoracic cavity (although a considerable quantity had been removed), with adhesions and organization of the exudate. The pleura and mediastinum were enormously thickened with a ruptured abscess between the posterior mediastinum. Disease further advanced in right side. On examining lymphatic glands of the affected limb nothing abnormal could be found with the naked eye. In the saphena vein was found a thrombus which could be felt forming two days previous to death, about two ounces in weight. Also in each lung was found an embolus—one about four ounces and the other about two ounces in weight. Not one parasite could be found, but the walls of the small intestines were considerably thickened.

Another typical case of embolus occurred in the practice of a neighboring veterinarian, Dr. Orr, of Baden, a case to which I was called in consultation some months ago, and pronounced lymphangitis, or, in my opinion, the result of thrombus. The case improved, and about two weeks previous to death, which occurred about October 1st, showed difficulty in breathing. Veterinary aid was not called, and the animal was found dead in the pasture. Dr.

Orr held a post-mortem and showed me what he found: An embolus in right auricle about two to four ounces in weight, and two others from the pulmonary arteries from the bifurcation, each weighing as much as the one from the heart. The right kidney was of enormous size, the capsule distended and filled with gelatinous substance. The enormous mass was divided as near half as possible, and one-half was brought for me to see, and this, when put on a scale with the suprarenal capsule, which was also enlarged, weighed twenty-four pounds.

The kidney trouble I cannot well account for, as the animal was not seen previous to death, unless it was a result of the impure state of the blood occurring through pulmonary obstruction. But if such was the cause, I would expect both kidneys to be in the same condition. I may say that a portion of the affected kidney to the naked eye seemed in fairly good condition. I could not name the disease; and would like to be informed as to its nature, as I had only a lamp-light examination and my description is meagre. It appeared to me as if about one-half of the organ was changed and enlarged by straw-colored, gelatinous material taking the place of the proper kidney substance, the capsule being over the whole mass and the interconnective tissue being distinctly visible on a cut surface (weight of the kidney alone about forty-five pounds). I did not see the left kidney, but understand that it looked normal. The thought also strikes me that if this kidney trouble had been of old standing it may have caused the abnormal state of blood, which led to thrombus and subsequently the embolus, which would be the direct cause of death from asphyxia, or, more properly, a non-oxygenated state of the blood from inability to gain entrance into the lungs.

